# Full-Length Transcriptome Analysis Identifies Candidate Molecular Features Associated with Golden-Lip Coloration in *Pinctada fucata martensii*

**DOI:** 10.3390/ani16192989

**Published:** 2026-09-23

**Authors:** Yong Liu, Shuai Chen, Hongmei Yang, Weilin Du, Wenhao Yu, Yuewen Deng, Zhe Zheng

**Affiliations:** 1 Fisheries College, Guangdong Ocean University, Zhanjiang 524088, China; liuyong_liufen@163.com (Y.L.); chsh304@stu.gdou.edu.cn (S.C.); m18218045816@163.com (H.Y.); dwl307@stu.gdou.edu.cn (W.D.); yuwenhao1@stu.gdou.edu.cn (W.Y.); dengyw@gdou.edu.cn (Y.D.); 2 Shenzhen Institute, Guangdong Ocean University, Shenzhen 518120, China; 3 Guangdong Provincial Key Laboratory of Aquatic Animal Disease Control and Healthy Culture, Zhanjiang 524088, China; 4 Pearl Research Institute, Guangdong Ocean University, Zhanjiang 524088, China; 5 Pearl Breeding and Processing Engineering Technology Research Centre of Guangdong Province, Zhanjiang 524088, China

**Keywords:** shell color, PacBio Iso-Seq, alternative splicing, biomineralization, *Pinctada fucata martensii*

## Abstract

Shell and pearl coloration is an economically important trait in *Pinctada fucata martensii*, with the golden-lip phenotype associated with commercially valuable golden nacre. In this study, full-length transcriptome sequencing was used to characterize transcript-abundance and transcript-structure patterns in pooled mantle samples from golden- and silver-lip oysters, with one pooled Iso-Seq library generated for each tissue/color condition from three individuals. Among the analyzed mantle samples, the largest transcript-profile differences between the color-line libraries were observed in mantle pallial tissue. The ferritin heavy-chain gene *fth1-a* showed moderately higher normalized transcript abundance in the Y_MP library than in W_MP but did not meet the predefined twofold screening threshold for library-level abundance differences. RT-qPCR analysis of additional independent individuals showed significantly higher *fth1-a* expression in golden-lip oysters. A custom anti-PmFTH1-A antibody was evaluated by Western blotting, and immunofluorescence analysis detected PmFTH1-A immunoreactivity within the decalcified shell organic matrix. Candidate alternative-splicing and alternative-polyadenylation events were identified in transcripts associated with carotenoid-related processes and biomineralization. *COL1A1* showed higher transcript abundance in Y_MP, and RT-qPCR analysis of additional individuals showed significantly higher *COL1A1* expression in the golden-lip group. Additional library-level differences involved transcripts associated with immune and stress-response processes. Overall, this exploratory study identifies candidate transcriptional and transcript-structural differences between the analyzed golden- and silver-lip samples and provides independent experimental support for higher *fth1-a* and *COL1A1* expression in golden-lip oysters. These candidates require functional investigation and validation in independently replicated populations before any evaluation as molecular markers for selective breeding.

## Simple Summary

Golden-lip oysters of *Pinctada fucata martensii* produce commercially valuable golden nacre and pearls, making coloration an important trait for pearl-quality improvement and selective breeding. However, the molecular features associated with this phenotype remain poorly characterized. In this study, full-length transcriptome sequencing was used to compare pooled mantle tissues from golden- and silver-lip oysters, with one sequencing library generated for each pooled tissue/color condition from three individuals. Among the analyzed samples, the largest transcript-profile differences were observed between the mantle pallial libraries. The ferritin gene *fth1-a* showed moderately higher normalized transcript abundance in the golden-lip library but did not meet the predefined twofold library-level screening threshold; RT-qPCR analysis of additional independent individuals nevertheless showed higher *fth1-a* expression in golden-lip oysters. PmFTH1-A immunoreactivity was detected in the decalcified shell organic matrix. *COL1A1* also showed higher transcript abundance in the Y_MP library, and this expression difference was independently supported by RT-qPCR. Candidate alternative-splicing and alternative-polyadenylation events were detected in transcripts associated with carotenoid-related processes and biomineralization. These findings provide candidate genes and transcript features for further functional investigation and validation in independent populations.

## 1. Introduction

Color polymorphism is widespread among marine and terrestrial organisms and is frequently targeted during domestication and selective breeding because of its economic value, visual distinctiveness, and ease of phenotypic identification [1]. In crops, livestock, and aquaculture species, color traits are often associated with nutritional quality, stress tolerance, and other performance-related characteristics [2,3]. In marine organisms, including fish, shrimp, and mollusks, color morphs have likewise been incorporated into breeding programs and evaluated alongside traits such as growth, survival, and environmental tolerance [4,5,6,7]. In some aquaculture species, coloration has also been examined in relation to other economically relevant or physiological traits, although such relationships are highly species- and phenotype-dependent.

Molluscan shell coloration is a complex phenotype shaped by multiple biochemical and structural components. It may arise from pigment deposition, including carotenoids, melanins, and porphyrin-related compounds, together with metal-associated components, shell-matrix proteins, calcium carbonate crystal organization, and shell microstructure [8,9]. At the molecular level, genomic variation, DNA methylation, transcriptional regulation, non-coding RNAs, and post-transcriptional processes may all contribute to color-associated phenotypic variation [10,11,12,13,14]. In bivalves, these regulatory layers have been investigated using genetic, transcriptomic, epigenetic, and biochemical approaches, although their relative contributions vary among species and color phenotypes [13,15,16,17,18,19,20]. Nevertheless, current evidence remains fragmented across regulatory levels and has focused largely on upstream variants, methylation profiles, candidate genes, and expression correlations. How these molecular differences relate to tissue-specific processes associated with visible color divergence remains incompletely understood.

Post-transcriptional transcript variation, particularly alternative splicing (AS) and alternative polyadenylation (APA), represents an additional regulatory layer through which transcript diversity may be associated with phenotypic variation. Emerging evidence from aquatic animals suggests that transcript-structure changes can accompany phenotypic variation [21,22]. For example, differential splicing of pigment-related genes has been associated with carotenoid deposition, and, in another molluscan system, a non-coding SNP affecting *ITGA8* splicing was experimentally linked to carotenoid-based coloration and tissue remodeling [23,24]. Long-read transcriptomic sequencing has also been applied in *P. f. martensii*, providing information on full-length transcripts, alternative splicing, lncRNAs, and transcript diversity [25]. However, isoform-level variation, AS, and APA in selectively bred pearl-oyster color lines have received limited investigation.

This question is especially relevant to the pearl oyster *Pinctada fucata martensii*, in which nacre color strongly influences the value and quality of cultured pearls [18,26]. Golden pearls are commercially important products in pearl aquaculture [27]. Unlike many bivalves, for which color studies focus mainly on the external shell, pearl oysters allow direct investigation of the nacreous layer that contributes substantially to pearl color and quality [26]. Previous studies in *P. f. martensii* have shown that golden-lip oysters contain higher levels of carotenoids and Fe^2+^ in the shell and that their mantle tissues show increased abundance or expression of genes and proteins associated with these components [28]. Nacre color has also been associated with DNA methylation differences between mantle pallial (MP) tissues of golden- and silver-lip oysters, while comparisons among mantle regions within golden-lip oysters suggest regional molecular heterogeneity [19]. Together, these observations suggest that color-associated molecular differences may also vary among mantle regions. However, whether transcript-structure variation is associated with nacre color differences remains unclear.

In this study, we performed PacBio Iso-Seq on pooled mantle edge (ME), mantle pallial (MP), and mantle center (MC) tissues from three golden-lip oysters and on pooled MP tissue from three silver-lip oysters, with one sequencing library generated for each tissue/color condition. By integrating transcript-abundance, AS, and APA analyses, we aimed to characterize transcript-level and transcript-structure patterns among the analyzed mantle regions and between the analyzed golden- and silver-lip pooled samples. Particular attention was given to candidate transcripts related to pigmentation, iron handling, and biomineralization.

## 2. Materials and Methods

### 2.1. Sample Collection

Adult *Pinctada fucata martensii* oysters were obtained from an oyster farm in the coastal area of Dajing Village, Zhanjiang, China. All sampled oysters were male, sexually mature, and in good health. The oysters were approximately 2 years old, with a mean shell height of 68.74 ± 6.18 mm, a mean shell length of 67.47 ± 6.70 mm, and a mean total weight of 45.92 ± 9.14 g. The oysters were farm-reared in coastal waters; at the time of sampling, the water temperature was 26.5 ± 0.5 °C and salinity was 30 psu. The golden-lip and silver-lip oysters represented selectively bred intraspecific color lines of the same species, characterized primarily by golden and silver nacre coloration, respectively [19]. For PacBio Iso-Seq analysis, three individuals were selected from each color line. For each tissue type, equal amounts of tissue from the three individuals of the same color line were pooled to generate one sequencing sample. Mantle edge (ME), mantle pallial (MP), and mantle center (MC) tissues were collected from golden-lip oysters to generate the pooled Y_ME, Y_MP, and Y_MC samples, respectively, whereas mantle pallial tissue from three silver-lip oysters was pooled to generate the W_MP sample (Figure 1a). Thus, each Iso-Seq library represented one pooled sample composed of three individuals rather than three independent biological replicates. Accordingly, comparisons between color lines should be interpreted as exploratory comparisons between pooled sequencing libraries and do not permit estimation of inter-individual variation or population-level statistical inference. All samples were immediately frozen in liquid nitrogen and stored at −80 °C until RNA extraction. In total, six oysters (three per color line) contributed to the Iso-Seq pools, and ten additional oysters (five per color line) were used for independent RT-qPCR validation, for a total of 16 biological individuals.

### 2.2. RNA Extraction, cDNA Synthesis, and Quantitative Real-Time PCR (RT-qPCR)

Total RNA was extracted using TRIzol^®^ reagent (Invitrogen; Thermo Fisher Sci-entific, Inc., Waltham, MA, USA). Genomic DNA was removed by treatment with 1 μL DNase I (Promega Corporation, Madison, WI, USA) at 37 °C for 15 min. RNA concentration and purity were assessed using a NanoDrop ND-1000 spectrophotometer (Thermo Fisher Scientific). For Iso-Seq library construction, RNA quality was further evaluated using an Agilent 2100 Bioanalyzer (Agilent Technologies, Inc., Santa Clara, CA, USA) with the RNA 6000 Nano Kit, including assessment of the RNA integrity number (RIN), 28S/18S ratio, and fragment-size distribution. For RT-qPCR, first-strand cDNA was synthesized from 1 μg of total RNA using oli-go(dT)-adaptor primers and gene-specific primers with M-MLV reverse transcriptase (Promega Corporation, Madison, WI, USA). The resulting cDNA was diluted fivefold before RT-qPCR analysis. For RT-qPCR validation, five additional golden-lip and five additional silver-lip oysters were collected from the same population described in Section 2.1. Primer sequences are provided in Table S1. RT-qPCR was performed in a total volume of 10 μL containing 5 μL of SYBR Green master mix, 0.4 μL of diluted cDNA, 0.4 μL of each primer (10 μM), and 3.8 μL of double-distilled water. The cycling conditions were 95 °C for 5 min, followed by 40 cycles of 95 °C for 10 s, 60 °C for 15 s, and 72 °C for 15 s. A melting-curve analysis was then performed at 95 °C for 10 s, 65 °C for 1 min, 95 °C for 1 s, and 37 °C for 30 s to assess amplification specificity. Primer amplification efficiencies were evaluated using fivefold serial dilutions of cDNA. The amplification efficiencies of the *fth1-a* and *gapdh* primer pairs were 93.7% and 94.6%, respectively, with corresponding R^2^ values of 0.9949 and 0.9853 (Figure S1). Each biological sample was analyzed in three technical replicates. The *gapdh* gene was used as the reference gene based on its previous use as an internal control in qRT-PCR studies of *P.f.martensii* [29]. Relative expression levels were normalized to *gapdh* and calculated using the 2^−ΔCt method. Statistical comparisons between the two phenotypes were performed using a *t*-test in GraphPad Prism 10.1.2.

### 2.3. Preparation of the Anti-PmFTH1-A Antibody and Immunofluorescence Analysis

A custom rabbit polyclonal antibody against PmFTH1-A was generated by Abmart Biomedical Co., Ltd. (Shanghai, China) using the synthetic peptide C-GGRIVLQDIKKPDRDDWGTGL as the immunogen. The tertiary structure of PmFTH1-A was predicted using SWISS-MODEL Workspace (https://swissmodel.expasy.org/; accessed on 15 May 2025) to assess the structural context of the selected antigenic peptide. The peptide region selected for antibody production was mapped onto the predicted three-dimensional structure to visualize its spatial location and surface exposure (Figure S2). Antibody reactivity was evaluated by Western blotting using mantle pallial protein extracts. Five mantle pallial protein samples were examined. Total proteins were extracted using RIPA lysis buffer supplemented with 1% PMSF, and protein concentrations were determined using the bicinchoninic acid (BCA) assay. Equal amounts of protein (30 μg per lane) were separated by 10% SDS-PAGE and transferred onto PVDF membranes (IPVH00010; Millipore, Billerica, MA, USA) at 300 mA for 90 min. Membranes were blocked with 5% non-fat dry milk in TBST for 1 h at room temperature and incubated overnight at 4 °C with the anti-PmFTH1-A antibody at a dilution of 1:1000. After washing, the membranes were incubated with an HRP-conjugated secondary antibody at a dilution of 1:10,000 for 1 h at room temperature. Immunoreactive bands were visualized using enhanced chemiluminescence. The predicted molecular weight of PmFTH1-A was approximately 25 kDa, and a clear major immunoreactive band was detected close to the expected molecular-weight region (Figure S2). This assay was performed for antibody-reactivity evaluation rather than quantitative comparison of PmFTH1-A abundance between phenotypes; therefore, no loading-control normalization or between-group densitometric analysis was performed. No dedicated positive or negative control was included in this Western-blot antibody-reactivity evaluation.

Shell organic matrix frameworks were prepared by EDTA decalcification. Shell pieces of approximately 0.8 × 1.2 cm containing both nacreous and prismatic layers were cleaned with ddH_2_O and air-dried. The pieces were partially embedded in paraffin, leaving the region to be decalcified exposed. The exposed shell region was then decalcified in 0.5 M EDTA (pH 8.0) containing 5% formaldehyde at room temperature for 3–5 days. After decalcification, the residual organic matrix framework was washed three times with ddH_2_O for 20 min each, immersed in liquid O.C.T. compound for 6 h, frozen, and cryosectioned at a thickness of 10 μm.

Immunofluorescence staining was performed using an SABC-Cy3 (rabbit IgG) immunohistochemistry kit (SA1074; Boster Biological Technology Co., Ltd., Wuhan, China) according to the manufacturer’s instructions with minor modifications. Sections were dried at 42 °C for 2 h and washed twice with ddH_2_O for 5 min each. Antigen retrieval was performed in citrate buffer (AR0024, Boster, Wuhan, China) at 100 °C for 1 min, followed by cooling to room temperature; this procedure was repeated three times. Sections were blocked with goat serum for 20 min at room temperature and incubated with the rabbit anti-PmFTH1-A primary antibody at a dilution of 1:20 at 37 °C for 1–3 h. For the negative control, the primary antibody was omitted and replaced with the primary-antibody diluent, while all subsequent incubation procedures were performed identically to those used for the experimental sections. After washing with PBS, sections were incubated with biotinylated goat anti-rabbit IgG secondary antibody at a dilution of 1:100 at 37 °C for 0.5–1 h, followed by incubation with SABC-Cy3 reagent diluted 1:100 at 37 °C for 0.5–1 h in the dark. After final PBS washes, sections were mounted using antifade mounting medium (AR1109, Boster, Wuhan, China) and observed using a Nikon ECLIPSE Ni fluorescence microscope (Nikon Corporation, Tokyo, Japan) equipped with a DS-Ri2 camera (Nikon Corporation, Tokyo, Japan). Shell samples from three independent oysters per phenotype were examined, with at least three sections analyzed from each individual. Golden-lip and silver-lip shell sections were imaged under identical microscope and exposure settings, and representative images are shown. Immunofluorescence images were interpreted qualitatively for localization; no quantitative inference of protein abundance was made from fluorescence intensity.

### 2.4. PacBio Iso-Seq Library Preparation and Sequencing

Total RNA was subjected to mRNA enrichment using oligo(dT), followed by reverse transcription to generate cDNA. After end repair, A-tailing, purification, and ligation of circular adapters, circular sequencing libraries were generated; unligated fragments were removed by exonuclease digestion and the libraries were purified. Four pooled libraries (W_MP, Y_ME, Y_MP, and Y_MC) were sequenced on the PacBio Sequel platform.

### 2.5. PacBio Iso-Seq Data Processing and Full-Length Transcript Functional Annotation

PacBio sequencing reads were processed to obtain reads of insert (ROIs). Reads containing both 5′ and 3′ primer sequences together with a poly(A) tail were classified as full-length reads. Full-length reads were clustered using the cluster module of the official Iso-Seq3 pipeline and subsequently polished to generate high-quality consensus isoforms. The polished isoforms were aligned to the published *P. f. martensii* reference genome [30] (NCBI BioProject PRJNA283019; assembly GCA_002216045.1). Functional annotation was performed against the NT, NR, KOG, KEGG, Swiss-Prot, Pfam, and GO databases. BLASTN (v2.2.23) was used for NT annotation; BLASTX or DIAMOND (v0.8.31) was used for NR, KOG, KEGG, and Swiss-Prot annotation; and Blast2GO (v2.5.0) was used for GO annotation based on the NR results.

### 2.6. Prediction of Fusion Transcripts

Polished high-quality isoforms were aligned to the reference genome using GMAP (version 2020-06-02) with the parameters -t 24 -n 0. Predicted fusion transcripts were then identified using the fusion_finder.py script from the TOFU analysis suite (version 2.2.3) with default parameters.

### 2.7. Identification of Alternative Polyadenylation Sites

Alternative polyadenylation (APA) sites were identified using TAPIS v1.2.1 (-s 1) based on the genomic positions of the 3′ ends of aligned full-length isoforms and the reference GTF annotation. At least one trusted read was required by the analysis pipeline to support a poly(A) site; all other parameters were kept at their default settings. Because this permissive threshold can include false-positive or low-support sites, the resulting sites were treated as candidate APA events and interpreted descriptively rather than as statistically established differences between color lines.

### 2.8. Identification of Alternative Splicing Events

Alternative splicing (AS) and related transcript-structure events were identified from the polished full-length isoforms. Isoforms from all samples were first merged and aligned to the reference genome using GMAP, after which redundant isoforms were removed using tofu_collapse. The resulting non-redundant isoforms were compared with the reference gene annotation using cuffcompare in Cufflinks v2.0.2. ASprofile b-1.0.4 was then used with default parameters to catalogue exon skipping (SKIP and MSKIP), intron retention (IR and MIR), and alternative exon-end (AE) events. ASprofile also reports transcript start- and termination-site categories (TSS and TTS); these were retained as transcript-structure categories but were not interpreted as conventional splicing events. Events were catalogued separately for each library and compared descriptively. No biological-replicate-based statistical test of differential splicing or differential transcript-structure usage was performed.

### 2.9. Library-Level Abundance Comparison and Enrichment Analyses

Gene- and isoform-level abundances were quantified from the full-length Iso-Seq dataset. Full-length isoforms from all samples were merged and grouped according to genomic position. Sample-specific isoform counts were generated using the service provider’s original quantification pipeline and normalized against total isoform abundance within each library. The underlying in-house counting code was not publicly released by the service provider; this limitation is one reason the abundance comparisons are treated here as exploratory. PoissonDis was used by the original analysis pipeline to screen for library-level abundance differences, using fold change ≥ 2 and FDR ≤ 0.001 as computational screening thresholds. Because only one pooled sequencing library was available for each tissue/color condition, these FDR values do not estimate inter-individual biological variation and were not interpreted as evidence of population-level differential expression. Accordingly, genes and transcripts meeting these thresholds are referred to here as candidate library-level abundance differences rather than biologically replicated DEGs or DETs. GO and KEGG over-representation analyses were performed using the phyper function in R, with multiple-testing correction by FDR. FDR ≤ 0.01 was used to define statistically enriched terms or pathways; figures may also display top-ranked terms outside this cutoff for descriptive context, and these are not described as statistically significant.

### 2.10. Long Non-Coding RNA (lncRNA) Analysis

All assembled transcripts were evaluated for coding potential using CPC, txCdsPredict, CNCI, and Pfam-based screening. Transcripts were retained as high-confidence lncRNA candidates if they met the service provider’s combined non-coding criteria, had no significant Pfam protein-domain hit (E-value < 1 × 10^−5^), were >200 bp in length, and contained at least two exons. Because these assignments were computational, the resulting sequences are referred to as candidate lncRNAs. This analysis was used to characterize the full-length transcriptome and was not used to infer color-associated lncRNA regulation.

## 3. Results

### 3.1. High-Quality Full-Length Transcriptome Profiling Reveals Isoform Diversity and Predicted Fusion Transcripts in Mantle Tissues of Golden- and Silver-Lip P. f. martensii

PacBio SMRT sequencing of *Pinctada fucata martensii* mantle tissues generated a full-length transcript resource for transcript-structure analysis. Across the four libraries, 1,841,956 polymerase reads were generated, yielding 1,649,280 circular consensus sequence (CCS; Reads of Insert) reads. The mean insert length across libraries was 4349 bp, while the per-library mean insert lengths ranged from 4148 to 4592 bp (Table S2). Full-length non-chimeric reads were then identified and used for isoform clustering and polishing.

High-quality isoforms were mapped to the *P. f. martensii* reference genome. The overall mapping rate of polished isoforms exceeded 90%, whereas 72.73–79.75% showed unique genomic alignments; the difference reflects mapped isoforms with non-unique genomic alignments. Across libraries, 10,725–12,816 of the 27,512 annotated reference genes were detected, corresponding to detection rates of 38.98–46.58% (Table S3). The mean number of isoforms per detected gene ranged from 6.75 to 10.54 across libraries.

A total of 1808 predicted fusion transcript candidates were detected across the four mantle libraries, with 379–542 candidates per library (Table S4). W_MP contained 542 predicted fusion transcripts, compared with 389 in Y_MP; Y_MC and Y_ME contained 498 and 379, respectively. Most predicted fusion events were interchromosomal (92.0%), whereas intrachromosomal events accounted for 8.0% (Figure S3a–h). These fusion transcripts were identified computationally from the Iso-Seq data and were not experimentally validated in the present study.

### 3.2. Functional Annotation and lncRNA Prediction in Two Selectively Bred Color Lines of P. f. martensii

Of the 59,419 high-quality transcripts subjected to functional annotation, 34,469 (58.01%) were annotated in at least one of the NR, Pfam, KOG, Swiss-Prot, KEGG, and GO databases, while 3468 (5.84%) were annotated in all databases (Figure 1b; Table S5). NR-based homology analysis showed that the closest matches were predominantly to *Crassostrea gigas*, *Crassostrea virginica*, and *Mizuhopecten yessoensis* (Figure 1c). GO classification showed that cellular and metabolic processes were prominent biological-process categories, whereas binding and catalytic activity predominated among molecular functions. Transcripts annotated to biomineralization-, antioxidant-, and transcription-regulator-related functions were also identified (Figure 1d).

Coding-potential analysis classified 38,454 transcripts as candidate lncRNAs, 15,740 as mRNAs, and 5225 as unclear, accounting for 64.72%, 26.49%, and 8.79% of the analyzed transcripts, respectively (Figure 1e). Pfam screening further indicated that many candidate lncRNAs lacked recognizable protein domains (Figure 1f). Because these classifications were based on computational coding-potential predictions, the sequences are referred to as candidate lncRNAs rather than experimentally validated lncRNAs. These results are presented as transcriptome characterization and are not interpreted as evidence of color-associated lncRNA regulation.

### 3.3. Library-Level Gene and Transcript Abundance Profiles Across Color Phenotypes and Mantle Regions

Using the PoissonDis screening criteria defined in Section 2.9, 530 genes showed candidate library-level abundance differences in the W_MP versus Y_MP comparison, including 368 with higher abundance in W_MP and 162 with higher abundance in Y_MP. Y_MC versus Y_MP contained 183 such candidate genes (100 higher in Y_MC and 83 higher in Y_MP), whereas Y_ME versus Y_MP contained 117 (41 higher in Y_ME and 76 higher in Y_MP) (Figure S4a). These values describe differences between individual pooled sequencing libraries and are not estimates of population-level differential expression.

GO and KEGG analyses were used to summarize functional patterns among genes showing library-level abundance differences. In the W_MP versus Y_MP comparison, represented categories included cellular and metabolic processes, immune-system processes, cytoskeletal functions, and biological adhesion, while top-ranked KEGG pathways included Ribosome, Oxidative phosphorylation, Lysosome, Phagosome, Apoptosis, and Tight junction (Figure 2a,b). Only terms or pathways meeting the FDR ≤ 0.01 criterion were treated as statistically enriched; additional plotted categories are shown for descriptive context.

The normalized Iso-Seq dataset showed approximately 1.24-fold higher *fth1-a* transcript abundance in Y_MP than in W_MP (Figure 3a). This difference did not meet the predefined twofold threshold used for library-level abundance screening and was therefore not classified as a threshold-meeting candidate differential-abundance signal. Nevertheless, because ferritin was biologically relevant to the study question, *fth1-a* was examined independently by RT-qPCR in additional oysters. RT-qPCR analysis (*n* = 5 per phenotype) showed significantly higher *fth1-a* expression in golden-lip oysters than in silver-lip oysters (*p* < 0.001; Figure 3b). The difference in effect size between the Iso-Seq and RT-qPCR datasets was not interpreted quantitatively because the assays used different normalization procedures and independent biological samples.

To examine PmFTH1-A at the protein level, the custom rabbit polyclonal antibody was evaluated by Western blotting and then used for qualitative immunofluorescence localization. Western blot evaluation using five mantle pallial protein samples revealed a clear major immunoreactive band close to the predicted molecular-weight region of approximately 25 kDa (Figure 3c). This assay was used to evaluate antibody reactivity and was not used for quantitative comparison between golden- and silver-lip oysters. Immunofluorescence analysis of the decalcified shell organic matrix revealed PmFTH1-A-positive Cy3 signal in the shell matrix (Figure 3d). Three independent oysters per phenotype were examined, with at least three sections analyzed from each individual, and representative images are shown. Because fluorescence intensity was not quantitatively analyzed across biological replicates, the images are interpreted only as qualitative localization evidence. No obvious Cy3 signal was observed in the negative control.

Together, the RT-qPCR experiment independently supports higher *fth1-a* expression in the additional golden-lip oysters analyzed, whereas the immunofluorescence experiment demonstrates PmFTH1-A immunoreactivity within the decalcified shell organic matrix. These two observations are treated as complementary but distinct lines of evidence.

At the transcript level, W_MP versus Y_MP contained 587 transcripts meeting the library-level screening criteria, including 411 with higher abundance in W_MP and 176 with higher abundance in Y_MP. Y_MC versus Y_MP contained 217 such transcripts (109 higher in Y_MC and 108 higher in Y_MP), whereas Y_ME versus Y_MP contained 124 (42 higher in Y_ME and 82 higher in Y_MP) (Figure 4a,d and Figure S5a). Functional analyses of the W_MP versus Y_MP candidate transcript set included immune-related, antioxidant, and cytoskeletal categories, together with KEGG pathways such as Phagosome, Lysosome, Antigen processing and presentation, and Chemical carcinogenesis–reactive oxygen species (Figure 4b,c).

For Y_ME versus Y_MP, represented functional categories included biomineralization-related annotations, immune processes, biological adhesion, and Notch signaling, whereas Y_MC versus Y_MP included ATP-dependent activity, actin-cytoskeleton regulation, and metabolic pathways (Figure 4e,f and Figure S5b,c). These analyses describe functional patterns among transcripts with library-level abundance differences and should not be interpreted as biologically replicated regional effects.

### 3.4. Candidate Transcript-Structure Events Detected Across Pooled Libraries

#### 3.4.1. Global AS and APA Profiles

Global AS and APA analyses revealed extensive transcript-structure diversity in *P. f. martensii*. Most genes contained one or two detected APA sites, whereas a smaller subset contained multiple sites (Figure 5a–d).

After redundancy removal, 54,362 non-redundant transcripts were obtained, of which 50,423 were assigned to defined transcript categories. These included 1324 known transcripts, 30,917 splice variants of annotated genes, and 18,182 novel transcripts; the remaining 3939 belonged to other transcript categories (Figure 5e). Conventional AS categories included exon skipping, intron retention, and alternative exon ends. ASprofile also reported TSS/TTS-related transcript-structure categories, which were abundant in the dataset but are not interpreted here as conventional splicing events (Figure 5f,g; Table S6). W_MP contained more detected events in several categories than the golden-lip libraries. Among the three pooled golden-lip mantle libraries, the number of detected events varied descriptively as Y_MP > Y_MC > Y_ME; no biological-replicate-based test of regional differences was performed.

#### 3.4.2. Candidate AS/APA-Related Transcript-Structure Patterns Observed Across the Analyzed Libraries

To improve traceability, the corresponding reference gene/locus identifiers are provided for each candidate shown in Figure 6. These identifiers refer to the annotated genomic loci rather than individual transcript isoforms. Candidate transcript-structure events involving *CHS*, *LysM4*, *LDLR*, *HIF*, and *COL1A1* were detected in the W_MP and Y_MP libraries, together with a candidate APA event involving an EF-hand calcium-binding protein (Figure 6a,b). The corresponding Iso-Seq transcripts differed in exon structure and/or 3′-end position. Because only one pooled library was available per condition, these observations are interpreted descriptively as library-level transcript-structure patterns. Among the selected transcripts shown in Figure 7, normalized transcript abundance differed between the W_MP and Y_MP libraries. *COL1A1* showed higher normalized transcript abundance in Y_MP than in W_MP (Figure 7a). Independent RT-qPCR analysis showed significantly higher overall *COL1A1* expression in golden-lip oysters than in silver-lip oysters (*n* = 5 per phenotype, *p* < 0.0001; Figure 7b). This RT-qPCR result supports the overall expression difference in *COL1A1* but does not validate the specific AS event detected by Iso-Seq.

The pooled Y_ME and Y_MP libraries, each generated from the corresponding tissues of the same three golden-lip oysters, were also compared descriptively. *VWA* and a collagen-related transcript showed higher normalized abundance in Y_MP, whereas the nacre serine protease inhibitor *NSPI3* showed lower abundance; the other transcripts examined were detected in both libraries (Figure S6; Table S7). *LysM6* showed lower abundance in Y_MP, whereas the EF-hand calcium-binding protein, *NUSP3*, and mantle gene 7 showed higher abundance (Figure 6b,c; Figure 7g–j). These observations document library-level transcript-abundance and transcript-structure patterns and do not establish population-level regional differences.

## 4. Discussion

*Pinctada fucata martensii* exhibits variation in shell coloration and shell-layer composition. Nacre is particularly relevant because it directly influences pearl value [18,27]. In the present dataset, the largest transcript-profile differences among the analyzed comparisons were observed between the pooled golden- and silver-lip MP libraries. Because only one pooled library was available per condition, this comparison is exploratory and provides candidate molecular observations rather than population-level color-line effects.

Previous studies have reported higher carotenoid levels and elevated shell Fe^2+^ in golden-lip oysters, together with differences in iron- and lipid-associated proteins in mantle pallial tissue [19,28]. Ferritin is a conserved iron-storage protein that regulates intracellular iron availability [31,32], and ferritin-related genes have previously been implicated in pearl-oyster shell formation [33,34]. In the present Iso-Seq dataset, *fth1-a* showed only a moderate increase in normalized abundance in Y_MP relative to W_MP and did not meet the predefined twofold library-level screening threshold. Importantly, RT-qPCR in an independent set of oysters showed significantly higher *fth1-a* expression in the golden-lip group. Western blot evaluation using five mantle pallial protein samples revealed antibody reactivity close to the predicted molecular-weight region of PmFTH1-A (~25 kDa), while immunofluorescence localized PmFTH1-A-associated signal within the decalcified shell organic matrix. Three independent oysters per phenotype were examined for immunofluorescence, with at least three sections analyzed from each individual. These protein-level observations provide antibody-reactivity and localization evidence but do not demonstrate altered iron metabolism or a quantitative protein-abundance difference between color lines. Because iron content was not measured in the present study, a functional link between PmFTH1-A, iron handling, and golden nacre coloration remains a hypothesis for future testing.

The Iso-Seq dataset also identified candidate transcript-structure events in genes of potential relevance to coloration and biomineralization. In our previous study, SNPs in *LDLR* were associated with nacre color variation in *P. f. martensii* [35]. Here, a candidate *LDLR* APA-derived shortened transcript was detected and showed higher normalized abundance in the Y_MP library, whereas the overall gene-level comparison did not show the same pattern. Because the present design lacks biological replication for Iso-Seq and the shortened transcript was not experimentally validated, its stability, coding capacity, translation, and biological relevance remain unknown. The observation is therefore treated as a hypothesis-generating transcript feature rather than evidence of a functional APA mechanism.

Several biomineralization-related genes, including *CHS*, *LysM4*, *COL1A1*, and an EF-hand calcium-binding protein, also showed candidate transcript-structure variation in the analyzed libraries. *CHS* and EF-hand calcium-binding proteins have established links to chitin-based shell framework formation and calcium-related biomineralization [36,37], and shell-matrix, collagen-related, and calcium-binding proteins are well represented in molluscan biomineralization research [30,36,37,38,39,40]. *COL1A1* showed higher normalized transcript abundance in the Y_MP library, and RT-qPCR in additional individuals showed significantly higher overall *COL1A1* expression in golden-lip oysters. Because the RT-qPCR assay measured overall *COL1A1* expression rather than a specific isoform, it does not validate the AS event detected by Iso-Seq. These findings therefore support *COL1A1* as a candidate associated with shell-matrix differences, while the functional significance of the specific transcript structures remains to be tested.

Regional comparisons among mantle tissues were also exploratory. The pooled Y_MC and Y_MP libraries showed fewer library-level abundance differences than the Y_ME and Y_MP comparison, and represented functional categories were mainly related to basic cellular processes. Because each regional library was generated by pooling corresponding tissues from the same three golden-lip oysters, these observations cannot establish general regional specialization or estimate inter-individual variability. Additional independently replicated sampling is required to determine whether the observed patterns are reproducible across individuals.

The descriptive Y_ME versus Y_MP comparison identified candidate abundance and transcript-structure patterns involving biomineralization-related, immune-related, adhesion, and signaling annotations. These patterns are broadly compatible with known functional differences between mantle regions during shell formation [38,41,42,43,44], but the present data should be viewed as hypothesis-generating because the regional comparisons are based on single pooled libraries. Accordingly, the observed library-level differences do not by themselves demonstrate regional specialization at the population level.

A *HIF*-related isoform difference was also detected between the analyzed MP libraries. Previous studies in *P. f. martensii* and other oysters have reported color-line differences in antioxidant or stress-related traits [45,46,47]. However, no stress challenge, *HIF* activity, glycolysis, or physiological stress traits were measured in the present study. The *HIF*-related transcript observation is therefore presented only as a candidate for future investigation and does not provide evidence that golden-lip oysters have enhanced stress tolerance.

Several limitations should be considered when interpreting these results. Each PacBio Iso-Seq library was generated from tissues pooled from three individuals, but only one pooled library was available per tissue/color condition; therefore, the design did not provide independent biological replication for the Iso-Seq comparisons or permit estimation of inter-individual variation. The golden- and silver-lip oysters are selectively bred color lines, so broader genetic-background differences between the lines could also contribute to the observed library-level transcript patterns independently of coloration. The study was restricted to sexually mature male oysters sampled from the recorded farm environment, so generalizability across sex, reproductive stage, age, and environmental conditions remains untested. RT-qPCR provided independent biological replication only for *fth1-a* and *COL1A1*. The PmFTH1-A Western blot was used for antibody evaluation, and the immunofluorescence data were interpreted qualitatively for localization rather than as quantitative evidence of differential protein abundance. Most other candidate transcript features remain without direct functional validation, and pigment and metal contents were not measured in the present study.

Taken together, the present study identifies candidate library-level transcript patterns involving genes related to iron handling, pigment-associated processes, and biomineralization. *fth1-a* and *COL1A1* received independent support from RT-qPCR, whereas the AS/APA-related observations remain computational candidates detected in pooled libraries. These results provide hypotheses for future work on nacre coloration and shell-matrix biology. Evaluation of any candidate as a molecular marker will require larger independently sampled populations, direct phenotype measurements, and, where feasible, functional validation.

## 5. Conclusions

This exploratory study identified library-level transcriptional and transcript-structural patterns in pooled mantle samples from golden- and silver-lip *Pinctada fucata martensii*. Each Iso-Seq library was generated from tissues pooled from three individuals, but only one pooled library was available per condition. In the Iso-Seq dataset, *fth1-a* showed moderately higher abundance in Y_MP but did not meet the predefined twofold screening threshold; independent RT-qPCR nevertheless showed higher *fth1-a* expression in additional golden-lip oysters. PmFTH1-A immunoreactivity was detected in the decalcified shell organic matrix, providing localization evidence rather than quantitative validation of the transcript difference. *COL1A1* showed higher normalized abundance in Y_MP and significantly higher overall expression by RT-qPCR in additional golden-lip oysters. Candidate AS/APA-related transcript structures were also detected in genes associated with carotenoid-related processes and biomineralization, but these events were not validated at the population level. Overall, the findings identify candidate associations involving iron-handling and shell-matrix-related processes that may contribute to golden nacre coloration and warrant validation in independently replicated populations.

## Figures and Tables

**Figure 1:**
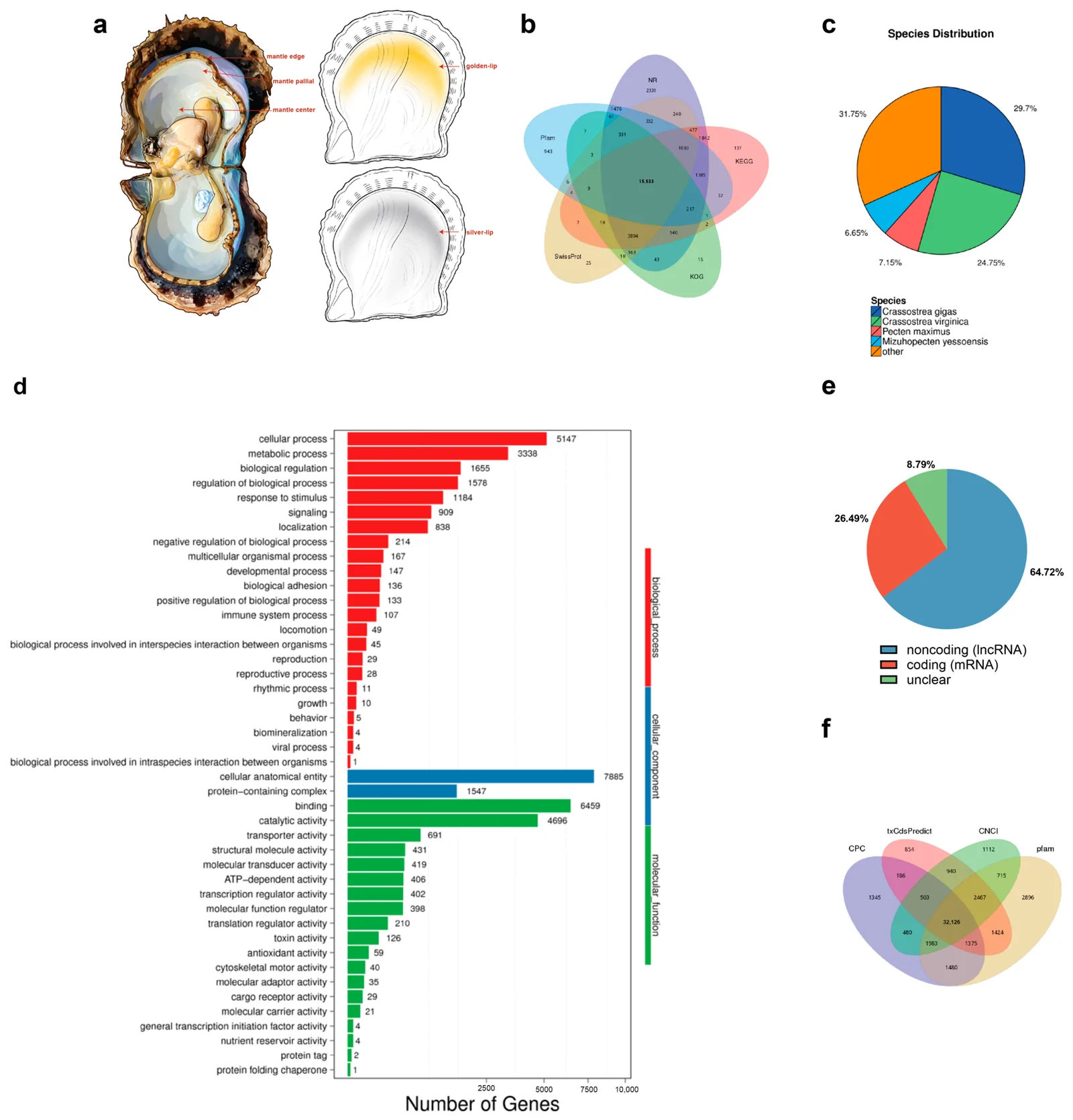
Sampling regions, functional annotation, and coding-potential analysis of full-length transcripts in *Pinctada fucata martensii*. (**a**) Representative golden-lip and silver-lip phenotypes and schematic illustration of the mantle sampling regions, including the mantle edge (ME), mantle pallial (MP), and mantle center (MC); (**b**) transcripts annotated in the NR, KOG, KEGG, Pfam, and Swiss-Prot databases; (**c**) distribution of homologous species identified in the NR database; (**d**) GO classification of putative functions of unique transcripts; (**e**) distribution of predicted transcript coding capacity; and (**f**) candidate lncRNAs predicted using CPC, CNCI, txCdsPredict, and Pfam. Non-overlapping regions indicate lncRNAs identified by a single tool, whereas overlapping regions indicate lncRNAs identified by multiple tools.

**Figure 2:**
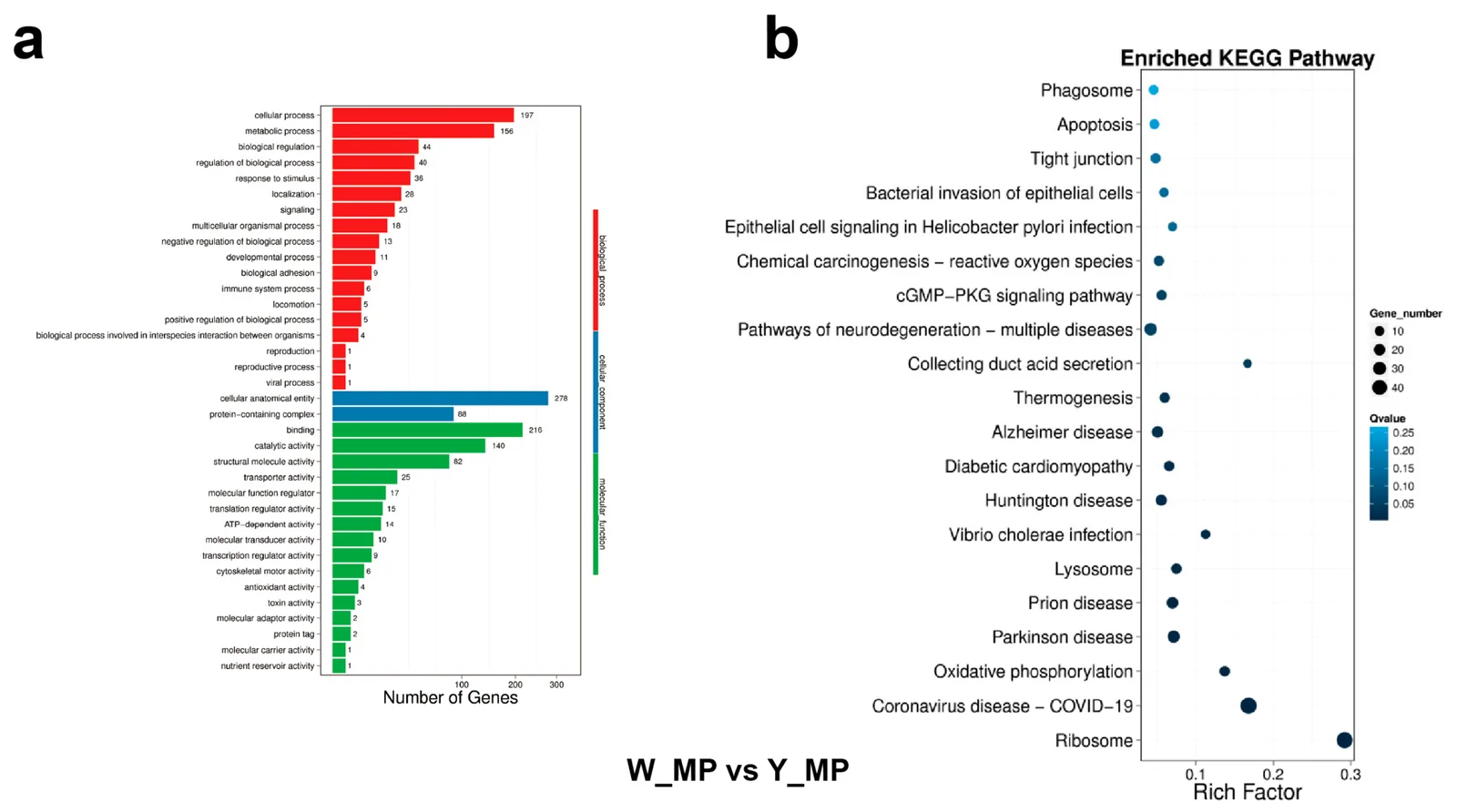
Library-level gene-abundance differences and functional over-representation in mantle pallial tissue. (**a**) GO analysis of genes showing library-level abundance differences in W_MP versus Y_MP; (**b**) KEGG pathway analysis of the same candidate gene set. Point size represents the number of genes, Rich Factor represents the ratio of candidate genes to all annotated genes in the corresponding pathway, and Q-values represent FDR-adjusted enrichment *p*-values. Because one pooled library was available per condition, these analyses are exploratory and do not provide population-level differential-expression inference.

**Figure 3:**
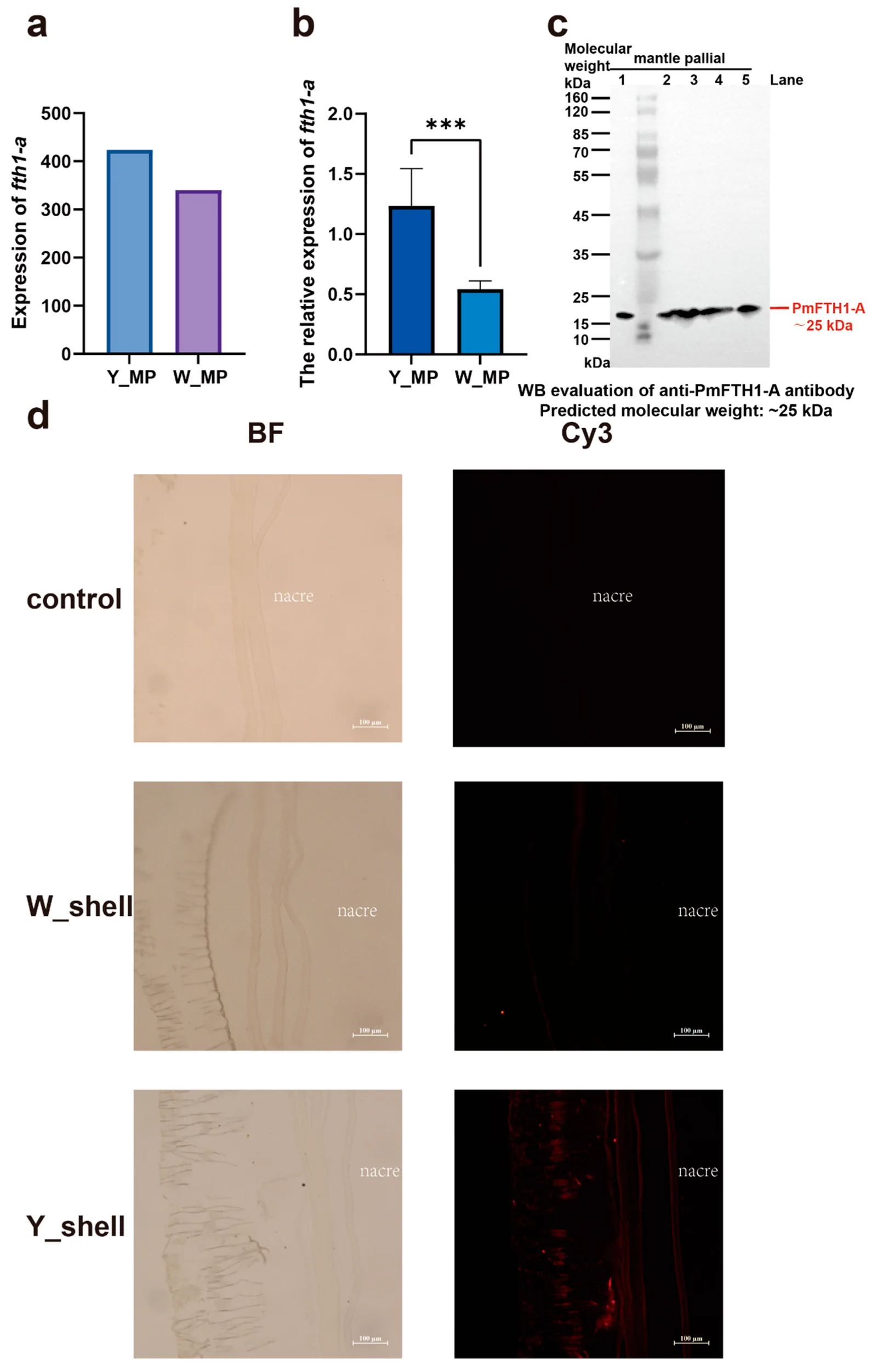
Expression analysis and immunolocalization of PmFTH1-A in golden- and silver-lip *Pinctada fucata martensii*. (**a**) Normalized fth1-a transcript abundance in the pooled Y_MP and W_MP Iso-Seq libraries; the approximately 1.24-fold difference is below the predefined twofold library-level screening threshold. (**b**) RT-qPCR analysis of fth1-a expression in independent mantle pallial samples from golden- and silver-lip oysters (*n* = 5 per phenotype). Data are presented as mean ± SD. Statistical significance was assessed using a *t*-test in GraphPad Prism 10.1.2. *** *p* < 0.001. (**c**) Western blot evaluation of the custom rabbit polyclonal anti-PmFTH1-A antibody using five mantle pallial protein samples. A clear major immunoreactive band was detected close to the predicted molecular-weight region of PmFTH1-A (~25 kDa). This assay was used to evaluate antibody reactivity rather than for quantitative comparison between phenotypes; no dedicated positive or negative control was included. (**d**) Immunofluorescence localization of PmFTH1-A in the decalcified shell organic matrix framework. Shell samples from three independent oysters per phenotype were examined, with at least three sections analyzed from each individual. Representative images are shown. For the negative control, the primary antibody was omitted and replaced with the primary-antibody diluent. BF, bright-field images; Cy3, PmFTH1-A-positive immunofluorescence signal; Control, negative control; W_shell, silver-lip shell; Y_shell, golden-lip shell; nacre, nacreous layer. Golden- and silver-lip sections were imaged under identical acquisition settings. Scale bars = 100 μm.

**Figure 4:**
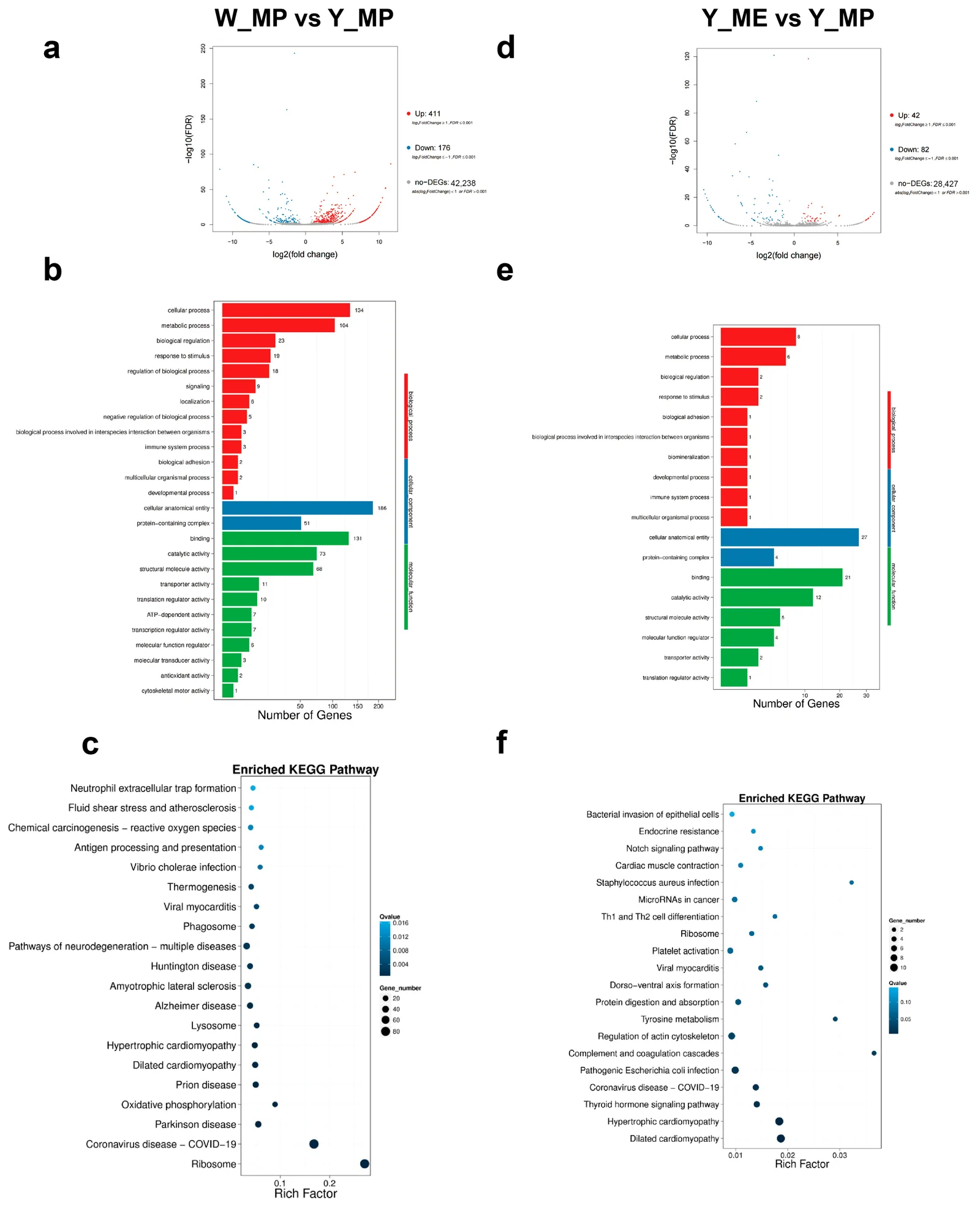
Library-level transcript-abundance differences and functional analyses in the W_MP versus Y_MP and Y_ME versus Y_MP comparisons. (**a**,**d**) Numbers of transcripts meeting the predefined library-level screening criteria; (**b**,**e**) GO analyses; and (**c**,**f**) KEGG pathway analyses. In the KEGG plots, point size represents the number of transcripts, Rich Factor represents the ratio of candidate transcripts to all annotated transcripts in the corresponding pathway, and Q-values represent FDR-adjusted enrichment *p*-values. These comparisons are exploratory because one pooled library was available for each condition.

**Figure 5:**
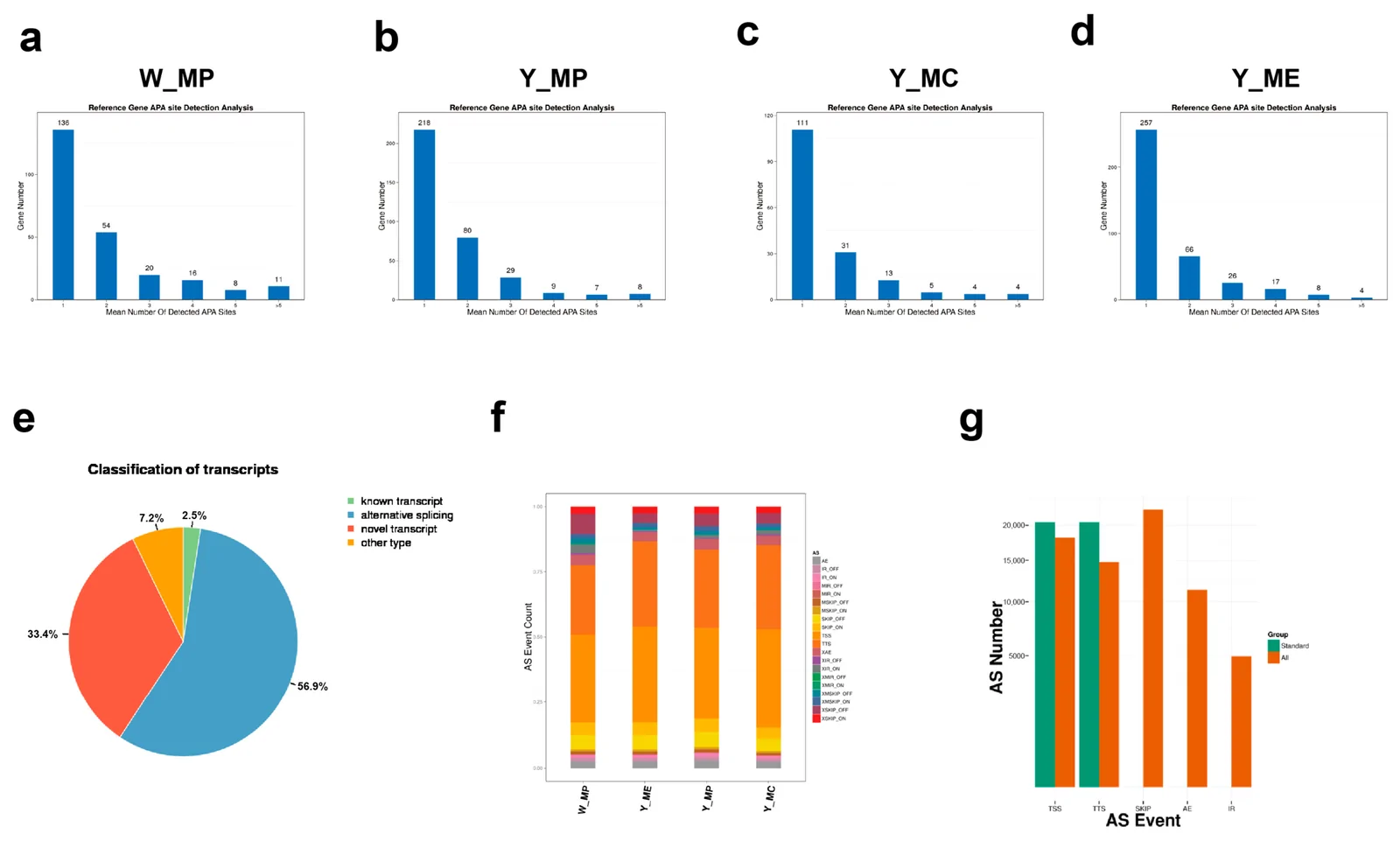
Characterization of alternative-polyadenylation (APA), conventional alternative-splicing (AS), and related transcript-structure events. (**a**–**d**) Distribution of the number of candidate APA sites identified per gene in each library; (**e**) transcript classification; (**f**) numbers of detected transcript-structure events; and (**g**) comparison between reference-annotated (known) and Iso-Seq-detected transcript-structure events. “Standard” denotes events present in the reference annotation, whereas “Alt” denotes events detected in the present Iso-Seq dataset. TSS, alternative transcription start site; TTS, alternative transcription termination site; SKIP, exon skipping; AE, alternative exon end; IR, intron retention.

**Figure 6:**
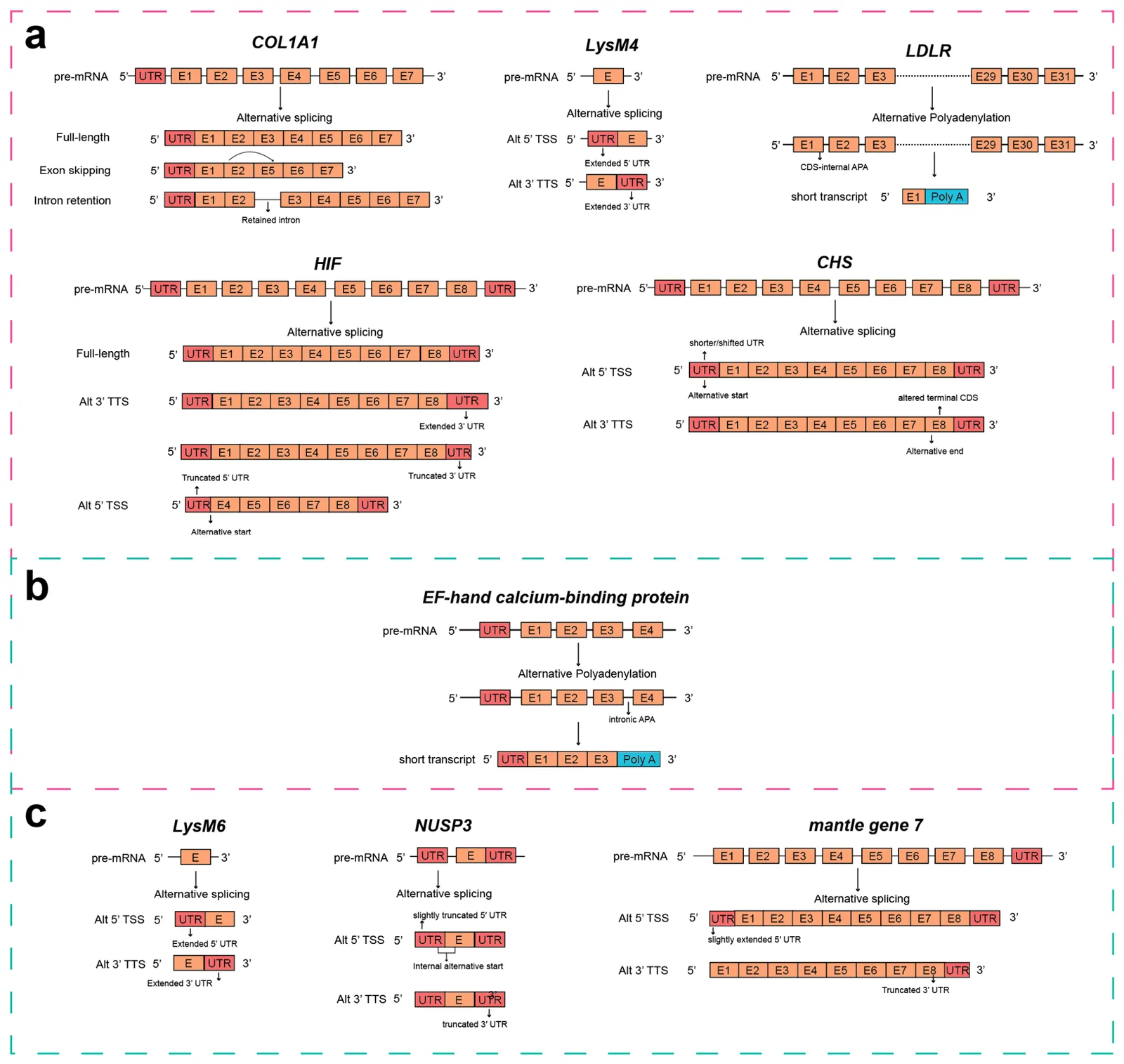
Schematic representation of candidate transcript-structure events detected in the analyzed pooled Iso-Seq libraries. Corresponding reference gene/locus identifiers are provided for each candidate: COL1A1 (PIN_scaffold_8_0867), LysM4 (PIN_scaffold_14_0701), LDLR (PIN_scaffold_1_0701), EF-hand calcium-binding protein (PIN_scaffold_14_1130), CHS (PIN_scaffold_7_2269), mantle gene 7 (PIN_scaffold_12_1264), NUSP3 (PIN_scaffold_5_0963), LysM6 (PIN_scaffold_11_0342), and HIF (PIN_scaffold_9_1209). These identifiers correspond to reference genomic loci rather than individual transcript isoforms. The structures shown are computationally detected candidate transcript forms and should not be interpreted as statistically demonstrated differential isoform regulation. In the schematic transcript structures, orange boxes indicate exons, red boxes indicate untranslated regions (UTRs), and blue marks indicate poly(A) sites. (**a**) Candidate AS/APA-related structures involving *CHS*, *LysM4*, *LDLR*, *HIF*, and *COL1A1* in the W_MP/Y_MP analysis; (**b**) candidate APA structures involving the EF-hand calcium-binding protein; and (**c**) candidate transcript-structure events involving *NUSP3*, *LysM6*, and mantle gene 7 in the Y_ME/Y_MP analysis. These schematics represent computationally detected transcript structures and do not demonstrate differential AS/APA regulation at the population level.

**Figure 7:**
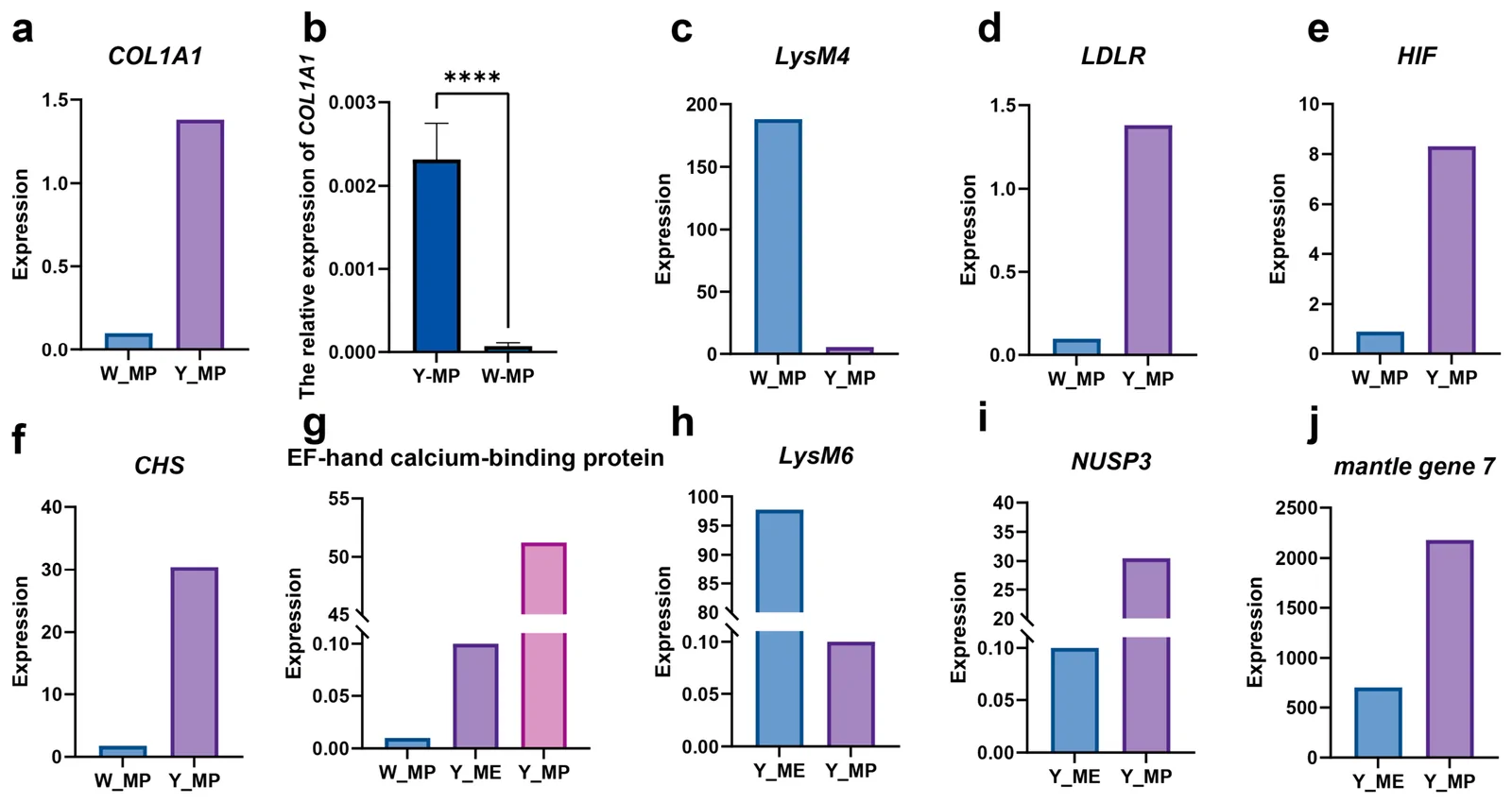
Expression patterns of selected color- and shell-formation-related transcripts and RT-qPCR validation of *COL1A1* in *Pinctada fucata martensii*. Normalized transcript abundance is shown for individual pooled Iso-Seq libraries: (**a**) *COL1A1* (PIN_scaffold_8_0867); (**c**) *LysM4* (PIN_scaffold_14_0701); (**d**) the candidate shortened *LDLR* transcript (PIN_scaffold_1_0701); (**e**) *HIF* (PIN_scaffold_9_1209); (**f**) *CHS* (PIN_scaffold_7_2269); (**g**) EF-hand calcium-binding protein (PIN_scaffold_14_1130), shown for W_MP, Y_ME, and Y_MP; (**h**) *LysM6* (PIN_scaffold_11_0342); (**i**) *NUSP3* (PIN_scaffold_5_0963); and (**j**) the uncharacterized transcript labeled “mantle gene 7” in the original analysis output (PIN_scaffold_12_1264), shown for Y_ME and Y_MP. These identifiers correspond to reference genomic loci rather than individual transcript isoforms. Each bar represents one pooled sequencing library; biological-replicate-based error bars and population-level statistical inference are therefore not applicable. PoissonDis screening thresholds, where used, were fold change ≥ 2 and FDR ≤ 0.001 and are interpreted only at the library level. (**b**) RT-qPCR analysis of overall *COL1A1* expression in independent mantle pallial samples from golden-lip and silver-lip oysters (*n* = 5 per phenotype). Relative expression levels were normalized to *gapdh* and calculated using the 2^−ΔCt method; no calibrator group was used. Data are presented as mean ± SD. **** *p* < 0.0001. Statistical significance for panel (**b**) was assessed using a *t*-test in GraphPad Prism 10.1.2.

## Data Availability

The original contributions presented in this study are included in the article. Further inquiries can be directed to the corresponding author.

## Supplementary Materials

The following supporting information can be downloaded at: https://www.mdpi.com/article/10.3390/ani16192989/s1, Figure S1: RT-qPCR amplification efficiency of *fth1-a* and *gapdh*. Standard curves were generated from serially diluted cDNA templates; Figure S2: Design information for anti-PmFTH1-A antibody preparation, predicted tertiary structure of the antigen, and Western blot evaluation of antibody reactivity; Figure S3: Characteristics of full-length transcript sequencing data and genomic distribution of fusion genes; Figure S4: Library-level gene-abundance differences and functional analyses among mantle samples; Figure S5: Library-level transcript-abundance differences and functional analyses between Y_MC and Y_MP; Figure S6: Expression patterns of selected nacre formation-related transcripts in *Pinctada fucata martensii*; Table S1: Primers used in this study; Table S2: Summary of PacBio SMRT sequencing data for *P. f. martensii* mantle tissues; Table S3: Genome and gene alignment statistics of *P. f. martensii* full-length transcripts; Table S4: Summary of predicted fusion transcripts identified in *P. f. martensii*; Table S5: The number of transcripts annotated in each database; Table S6: AS event statistics; Table S7: Candidate library-level transcript-abundance differences between Y_ME and Y_MP related to shell formation.
