# Supplementary material for: Full-Length Transcriptome Analysis Identifies Candidate Molecular Features Associated with Golden-Lip Coloration in Pinctada fucata martensii
Source: Animals (Basel). 2026 Sep 23;16(19):2989. doi: 10.3390/ani16192989 (PMC13663368; doi:10.3390/ani16192989)
Supplement: Supplementary file 1 [file animals-16-02989-s001.zip › Supplementary Figures.pdf]

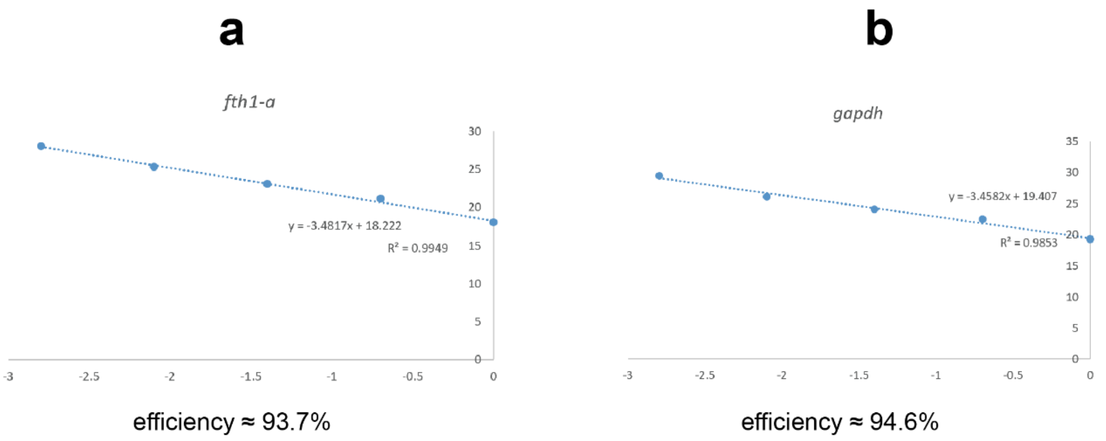

Figure S1. RT-qPCR amplification efficiency of *fth1-a* and *gapdh*. Standard curves were generated from serially diluted cDNA templates. Ct values were plotted against the log10-transformed relative template amount. (a) *fth1-a*, amplification efficiency = 93.7%, $R^2 = 0.9949$; (b) *gapdh*, amplification efficiency = 94.6%, $R^2 = 0.9853$.

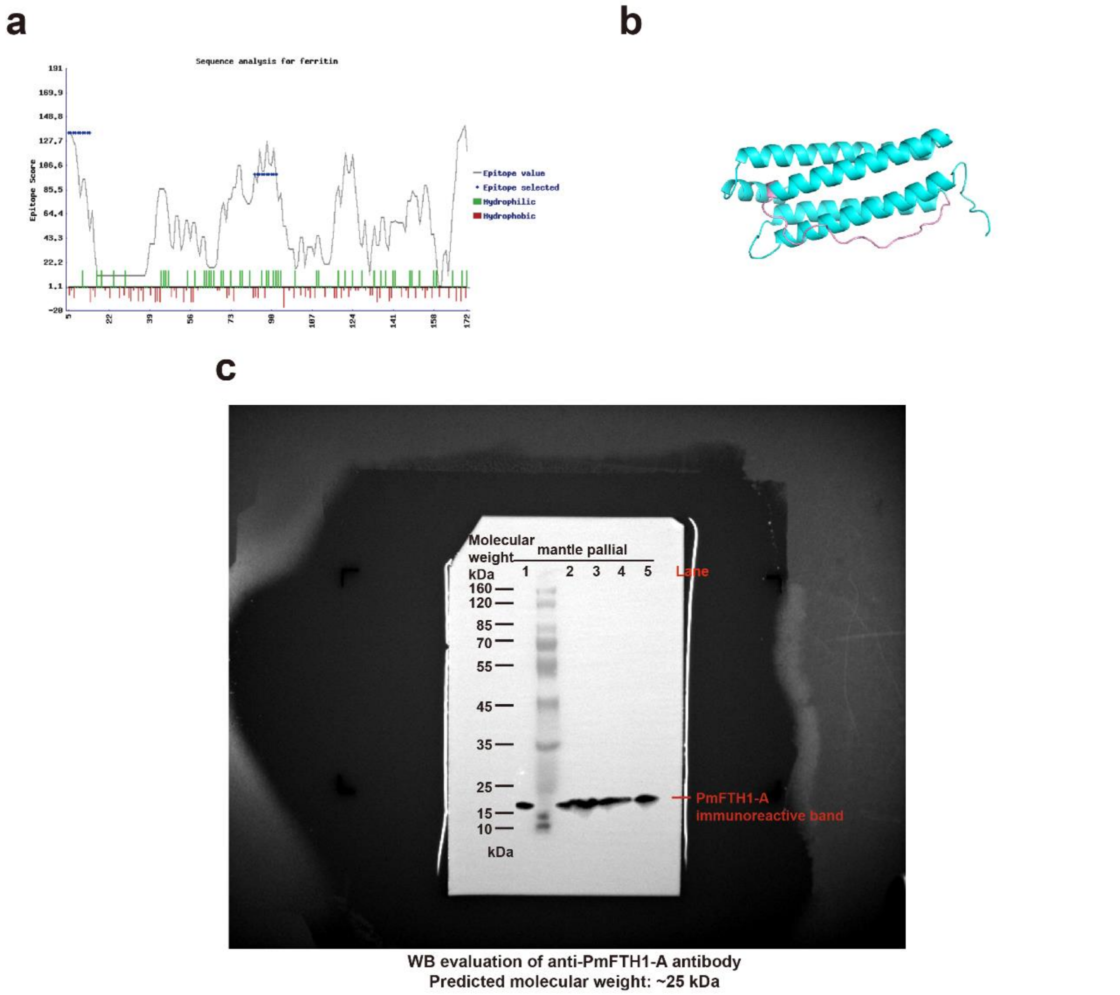

Figure S2. Design information for anti-PmFTH1-A antibody preparation, predicted tertiary structure of the antigen, and Western blot evaluation of antibody reactivity. (a) Antigen design information for PmFTH1-A. Red vertical lines indicate hydrophobic amino acid residues, green vertical lines indicate hydrophilic amino acid residues, and

purple diamonds indicate the selected epitope positions. (b) Tertiary structure of PmFTH1-A predicted using SWISS-MODEL. The antigenic peptide region used for antibody preparation is highlighted in red. (c) Western blot evaluation of the custom rabbit polyclonal anti-PmFTH1-A antibody using five mantle pallial protein samples. A clear major immunoreactive band was detected close to the predicted molecular-weight region of PmFTH1-A (~25 kDa). This assay was performed to evaluate antibody reactivity rather than for quantitative comparison of PmFTH1-A abundance between phenotypes; therefore, no loading-control normalization or between-group densitometric analysis was performed. No dedicated positive or negative control was included in this Western blot evaluation.

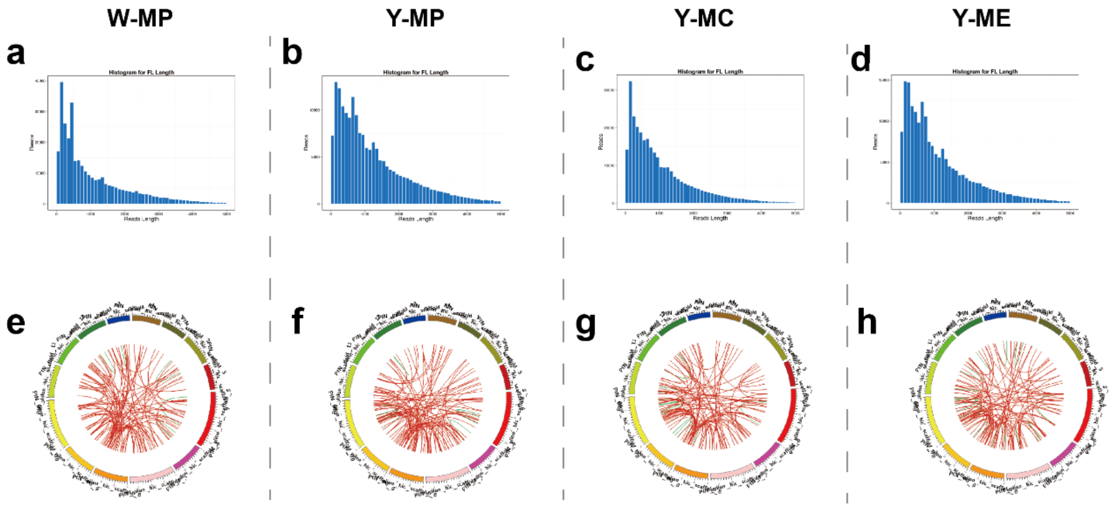

Figure S3. Characteristics of full-length transcript sequencing data and genomic distribution of fusion genes. (a-d) Density distribution of full length readsnon-chimeric (FLNC) read length obtained by SMART sequencing; (e-h) The distribution of identified fusion genes on the genome, Intra-chromosome data are coloured red inter-chromosome (green).

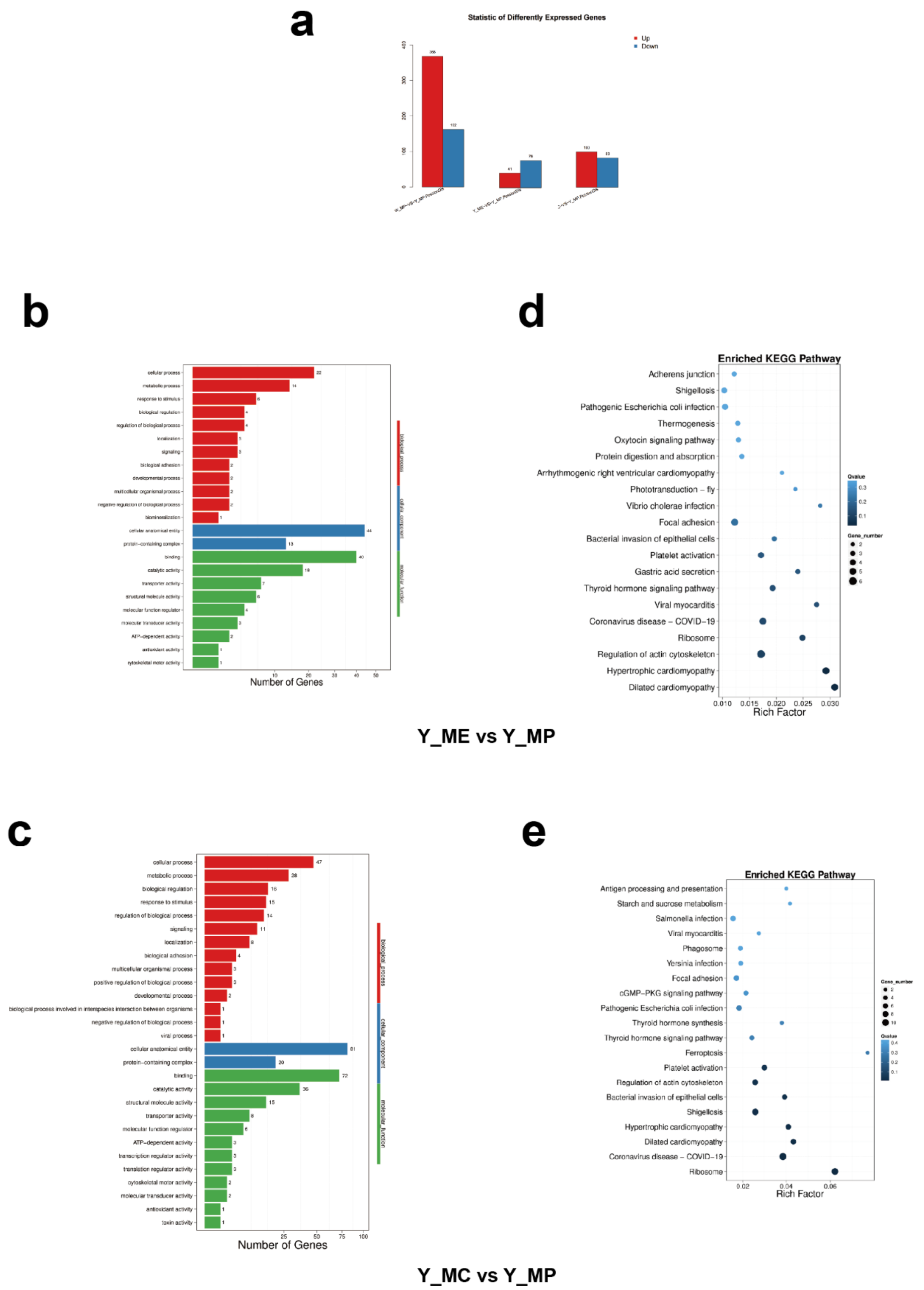

Figure S4. Library-level gene-abundance differences and functional analyses among mantle samples. (a) Genes showing candidate library-level abundance differences in the W_MP versus Y_MP, Y_ME versus Y_MP, and Y_MC versus Y_MP comparisons; (b,c) GO analyses of candidate genes in the W_MP versus Y_MP, Y_ME versus Y_MP, and Y_MC versus Y_MP comparisons, respectively; (d,e) KEGG pathway analyses of candidate genes in the Y_ME versus Y_MP and Y_MC versus Y_MP comparisons, respectively. Because only one pooled sequencing library

was available for each condition, these comparisons are exploratory and do not provide population-level differential-expression inference.

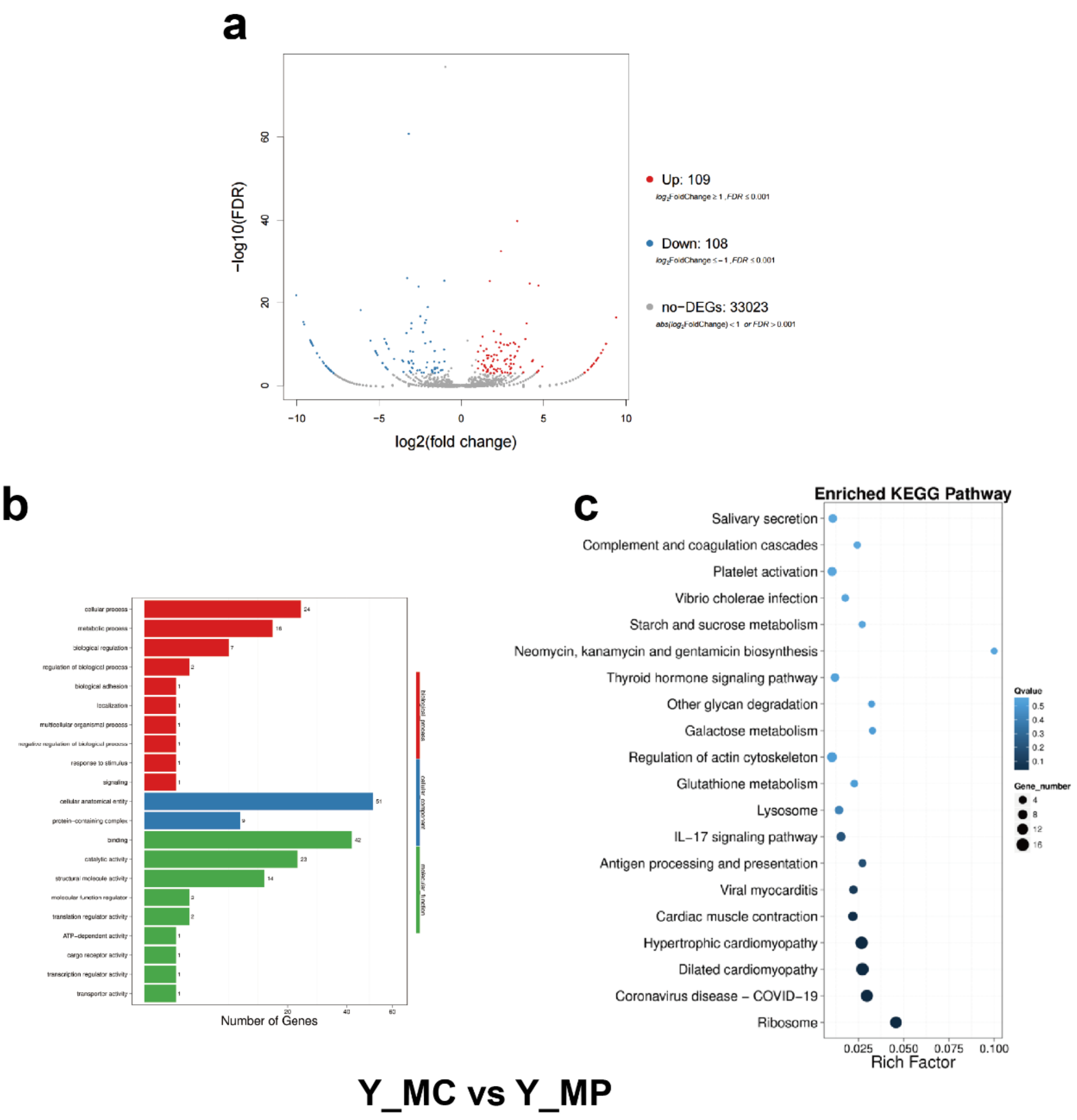

Figure S5. Library-level transcript-abundance differences and functional analyses between Y_MC and Y_MP. (a) Volcano plot of transcripts showing candidate library-level abundance differences between Y_MC and Y_MP; (b) GO analysis of these candidate transcripts; (c) KEGG pathway analysis of these candidate transcripts. Because only one pooled sequencing library was available for each condition, these comparisons are exploratory and do not provide population-level differential-expression inference.

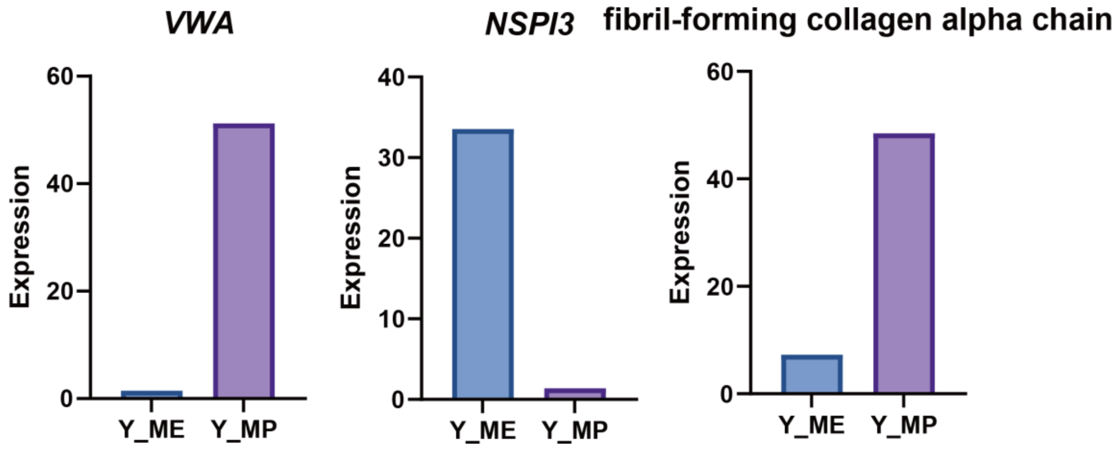

Figure S6. Expression patterns of selected nacre formation-related transcripts in *Pinctada fucata martensii*.
