# Supplementary material for: Full-Length Transcriptome Analysis Identifies Candidate Molecular Features Associated with Golden-Lip Coloration in Pinctada fucata martensii
Source: Animals (Basel). 2026 Sep 23;16(19):2989. doi: 10.3390/ani16192989 (PMC13663368; doi:10.3390/ani16192989)
Supplement: Supplementary file 1 [file animals-16-02989-s001.zip › Supplementary Tables.pdf]

Table S1 Primers used in this study.

Primer name	Primer sequence (5′ to 3′)
fth1-a-F	GATGATGTCGCTCTACCAGG
fth1-a-R	CAGGCTTCTTGATGTCTTGG
COL1A1-F	GGTGATAAGGGTGCACAAGGTTT
COL1A1-R	TTGGTCCAGGTTCTCCTTGTTGT
GAPDH-F	CACTCGCCAAGATAATCAACG
GAPDH-R	CCATTCCTGTCAACTTCCCAT

Table S2. Summary of PacBio SMRT sequencing data for *Pinctada fucata martensii* mantle tissues.

Samples	W_MP	Y_ME	Y_MP	Y_MC
Total Reads	474,822	459,365	425,160	482,609
CCS Reads	425,931	412,333	379,772	431,244
Mean Insert Length (bp)	4,400	4,255	4,592	4,148
FLNC Reads	194,715	109,300	111,517	175,474
Mean Isoform Length (bp)	1,418	1,469	1,569	1,333

Table S3. Genome and gene alignment statistics of *Pinctada fucata martensii* full-length transcripts.

Sample	W_MP	Y_ME	Y_MP	Y_MC
Input (FLNC reads)	194,715	109,300	111,517	175,474
Mapped (%)	93.91	90.62	91.93	91.72
Unmap (%)	6.09	9.38	8.07	8.28
Perfect map (%)	27.02	25.96	28.83	22.90
Unique map (%)	72.77	77.95	79.75	73.73
Multiple map (%)	21.14	12.67	12.18	17.99
Total RefGene	27,512	27,512	27,512	27,512

Sample	W_MP	Y_ME	Y_MP	Y_MC
Detected RefGene	12,816	11,993	10,725	10,909
Ratio of Total RefGene (%)	46.58	43.59	38.98	39.65
Mean isoforms per Gene	10.54	6.75	6.86	9.92

Table S4. Summary of fusion transcripts identified in *P. f. martensii*.

Sample	Total fusions	Inter-chromosomal	Intra-chromosomal	Mean coverage
W_MP	542	495 (91.3%)	47 (8.7%)	1.18
Y_MP	389	357 (91.8%)	32 (8.2%)	1.10
Y_ME	379	351 (92.6%)	28 (7.4%)	1.09
Y_MC	498	461 (92.6%)	37 (7.4%)	1.14
Total	1,808	1,664 (92.0%)	144 (8.0%)	1.13

Table S5. The number of transcripts annotated in each database.

Values	Number	Percentage
Total	59,419	100%
Nr	29,548	49.73%
Nt	13,734	23.11%
Swissprot	22,144	37.27%
KEGG	24,782	41.71%
KOG	20,456	34.43%

Values	Number	Percentage
Pfam	21,423	36.05%
GO	13,064	21.99%
Intersection	3,468	5.84%
Overall	34,469	58.01%

Table S6. AS event statistics.

AS Event Type	W_MP	Y_ME	Y_MP	Y_MC
AE	758	470	623	533
IR_OFF	373	223	277	250
IR_ON	322	207	298	222
MIR_OFF	43	30	32	35
MIR_ON	32	20	31	23
MSKIP_OFF	307	182	241	191
MSKIP_ON	238	158	195	167
SKIP_OFF	1,560	974	1,180	1,005
SKIP_ON	1,384	888	1,089	928
TSS	9,862	6,740	7,335	8,269
TTS	7,808	5,976	6,329	7,101
XAE	1,176	617	858	759
XIR_OFF	190	75	100	117
XIR_ON	929	94	214	346
XMIR_OFF	32	11	16	9

AS Event Type	W_MP	Y_ME	Y_MP	Y_MC
XMIR_ON	106	5	20	10
XMSKIP_OFF	586	174	314	240
XMSKIP_ON	476	323	400	323
XSKIP_OFF	2,255	664	990	867
XSKIP_ON	829	473	597	555

Abbreviations: SKIP, exon skipping; XSKIP, approximate exon skipping; MSKIP, multiple-exon skipping; XMSKIP, approximate multiple-exon skipping; IR, intron retention; XIR, approximate intron retention; MIR, multiple intron retention; XMIR, approximate multiple intron retention; AE, alternative exon end; XAE, approximate alternative exon end; TSS, alternative transcription start site; TTS, alternative transcription termination site. For paired event labels, "ON" denotes the transcript form containing the alternatively included exon or retained intron, whereas "OFF" denotes the corresponding form lacking that feature. The prefix "X" denotes an approximate event classification in ASprofile.

Table S7. Candidate library-level transcript-abundance differences between Y_ME and Y_MP related to shell formation.

Isoform ID	Gene name	NR ID	Species
PIN_scaffold_14_1130	EF-hand calcium-binding protein	ABF48089.1	*Pinctada fucata*
PIN_scaffold_5_0963	nacre uncharacterized shell protein 3	CCE46177.1	*Pinctada margaritifera*
PIN_scaffold_10_0428	von Willebrand factor type A,	XP_021351273.1	*Mizuhopecten yessoensis*
PIN_scaffold_12_1298	mantle gene 4	AAZ76258.1	*Pinctada fucata*
PIN_scaffold_3_0782	fibril-forming collagen alpha chain-like	XP_022341069.1	*Crassostrea virginica*
PIN_scaffold_13_0155	matrix metalloproteinase	BBE38636.1	*Pinctada fucata*
PIN_scaffold_12_1264	mantle gene 7	AAZ76261.1	*Pinctada fucata*
PIN_scaffold_12_1296	mantle gene 4	AAZ76258.1	*Pinctada fucata*
PIN_scaffold_12_1299	mantle gene 4	AAZ76258.1	*Pinctada fucata*

PB.12795.5	EF-hand calcium-binding protein	ABF48089.1	*Pinctada fucata*
PIN_scaffold_4_0407	mantle gene 8	AAZ76262.1	*Pinctada fucata*
PB.4039.3	nacre serine protease inhibitor 3	H2A0N9.1	*Pinctada margaritifera*
PB.3641.21	mantle gene 1	AAZ76255.1	*Pinctada fucata*
PB.4937.1	tyrosinase B3.2	AHZ34294.1	*Pinctada maxima*
PIN_scaffold_11_0342	lysine-rich matrix protein 3	BAH78728.1	*Pinctada fucata*
PIN_scaffold_10_0172	fibronectin type III domain-containing protein 1	XP_040584034.1	*Mesocricetus auratus*
